# Renal Function and Morphology in Aged Beagle Dogs Before and after Hydrocortisone Administration

**DOI:** 10.1371/journal.pone.0031702

**Published:** 2012-02-29

**Authors:** Pascale M. Y. Smets, Hervé P. Lefebvre, Luca Aresu, Siska Croubels, Hendrik Haers, Koen Piron, Evelyne Meyer, Sylvie Daminet

**Affiliations:** 1 Department of Small Animal Medicine and Clinical Biology, Faculty of Veterinary Medicine, Ghent University, Salisburylaan, Merelbeke, Belgium; 2 Université de Toulouse, INP, Ecole Nationale Vétérinaire de Toulouse, Unité de Recherche Clinique et Département des Sciences Cliniques, Toulouse, France; 3 Department of Public Health, Comparative Pathology and Veterinary Hygiene, Faculty of Veterinary Medicine, University of Padova, Viale dell'Università, Agripolis Legnaro PD, Italy; 4 Department of Pharmacology, Toxicology and Biochemistry, Faculty of Veterinary Medicine, Ghent University, Salisburylaan, Merelbeke, Belgium; 5 Department of Medical Imaging, Faculty of Veterinary Medicine, Ghent University, Salisburylaan, Merelbeke, Belgium; University of Georgia, United States of America

## Abstract

Objectives of this study were to evaluate glomerular filtration rate (GFR), renal structural changes and proteinuria in aged Beagle dogs before and after hydrocortisone (HC) administration. Eleven Beagle dogs ≥10 years old were treated with either hydrocortisone (HC group, n = 6) or placebo (control group, n = 5). Urinary markers, GFR and kidney biopsies were evaluated before (T0), during (T16 wks) and after discontinuing HC administration (T24 wks). Results indicate that HC administration causes a significant increase in GFR. At all time points except T16 wks, proteinuria was higher in the control group than in the HC group, and there was no significant difference in urinary markers between groups. At T16 wks, proteinuria, urinary albumin-to-creatinine (c) ratio, immunoglobulin G/c and retinol-binding protein/c were higher compared to baseline in the HC group. At T0, rare to mild renal lesions were detected in all HC dogs and rare to moderate changes in all control dogs. Glomerulosclerosis progressed in both groups until T24 wks. Tubular atrophy was detected in three HC dogs at T16 wks and T24 wks, but also in five control dogs throughout the study. At every time point, five HC dogs and all control dogs had rare to moderate interstitial inflammation. Rare to mild interstitial fibrosis was found in up to three HC dogs at T16 wks and T24 wks, and severe fibrosis in one HC dog at T24 wks. Up to four control dogs had rare to mild fibrosis at all time points. These findings indicate that clinically healthy, aged Beagle dogs may have considerable renal lesions and proteinuria, which could have implications for experimental or toxicological studies. Additional research is needed to elucidate glucocorticoid effects on renal structure, but functional changes such as hyperfiltration and proteinuria warrant attention to kidney function of canine patients with Cushing's syndrome or receiving exogenous glucocorticoids.

## Introduction

Proteinuria has been described in 44–75% of dogs with Cushing's syndrome and sometimes persists or even develops after successful treatment of hypercortisolism [1], [2]. An increased urinary albumin excretion has also been demonstrated in more than 80% of human Cushing patients prior to treatment of hypercortisolism [3]. Whereas short-term administration of exogenous glucocorticoids (GC) seems to increase GFR in humans and dogs, one study indicates a decreased glomerular filtration rate (GFR) in people with Cushing's syndrome and suggests that renal compromise in these patients warrants more attention [4], [5], [6]. Therefore, human and canine Cushing patients could be considered at risk for renal complications, but information about effects of GC on renal function is scarce.

Canine endogenous hypercortisolism or Cushing's syndrome is considered a good animal model for its human counterpart, because of similarities in the pathogenesis : 80–85% of cases are caused by a pituitary tumor and ACTH-dependent, while 15–20% are ACTH-independent [7]. Moreover, middle-aged to old individuals are typically affected in both species and similar clinical signs include fatigue, weight gain and central obesity with muscle atrophy of the limbs, polyuria, polydipsia and polyphagia, with the latter three being most pronounced in the dog [8], [9].

Previous experimental studies evaluating GC effects on renal function often differed from the *in vivo* situation in spontaneous Cushing's syndrome. For example, many experiments included young animals [10], [11]. Aging itself has marked effects on renal structure and function in humans, but also in laboratory animals, dogs and cats [12], [13], [14], [15], [16]. Therefore, in aged individuals GC will act on kidneys with an already decreased renal functional reserve [17], [18].

In addition to serum renal markers and GFR, urinary markers are gaining interest in veterinary medicine, because of their potential for early detection of renal dysfunction and ability to localize the insult to a particular part of the nephron [19], [20], [21]. These markers include high molecular weight (HMW) proteins such as immunoglobulin G (IgG), intermediate (I)MW proteins such as albumin (ALB), low (L)MW proteins such as retinol-binding protein (RBP) and urinary enzymes such as N-acetyl-β-D-glucosaminidase (NAG) [22], [23]. Glomerular dysfunction leads to higher filtration of ALB and in more advanced stages to presence of IgG in the ultrafiltrate [24]. Tubular dysfunction is reflected by urinary loss of RBP and NAG, due to disturbed reabsorption or increased protein trafficking and structural damage of tubule cells, respectively [25], [26].

Information about GC effects on renal function and morphology is rare, and often limited to case reports, despite their frequent therapeutic use and the occurrence of Cushing's syndrome in humans and dogs [11], [27], [28], [29], [30]. Therefore, the objectives of this study were to evaluate GFR, renal light microscopic and ultrastructural changes, and proteinuria in old Beagle dogs before and after administration of hydrocortisone.

## Materials and Methods

### Animals

This study was approved by the Ethical Committee of Ghent University (EC: 2008/146). Eleven female spayed Beagle dogs, age 8.4 to 11 years (median: 10 years), with a body weight ranging from 10.9 to 14 kg (median 12.9 kg) were included in the study. Dogs were housed in pairs in two adjacent indoor kennels of 2.6 m^2^ each, with free access to water, fed a standard dry food twice a day (Hill's science plan, Hill's Pet Nutrition, Breda, The Netherlands, 25.0% protein, 15.3% fat, 52.0% carbohydrates, 2.6% fiber), and allowed outdoor activity in a secured area. They were judged healthy based on physical examination, hematology, biochemistry profile, abdominal ultrasound and two consecutive morning urinary corticoid-to-creatinine ratio's (<10^*^10^−6^) to exclude endogenous Cushing's syndrome. A urinalysis consisting of sediment and dipstick analysis, urine specific gravity (USG), urinary protein-to-creatinine ratio (UPC), and bacterial culture were performed, but proteinuria (UPC>0.5) was not an exclusion criterion.

### Study design

Five dogs were randomly assigned to the placebo group (control group) and six to the hydrocortisone treatment group (HC group). After baseline sampling (T0), the control group received a gelatin capsule containing lactose and the HC-group received a capsule containing lactose and a median dose of 9.6 mg/kg HC (range from 8.7 to 11.0 mg/kg) every 12 hours (h). Hydrocortisone was used because it is the synthetic glucocorticoid most closely resembling endogenous cortisol and dosage was based on two previous publications [10], [11]. Dogs were monitored for development of clinical signs of GC excess such as polyuria, polydipsia and polyphagia, and for skin abnormalities using a scoring sheet. Treatment period was 16 weeks (end of treatment period: T16 wks), then the HC dose was tapered over 4 weeks (1 week 10 mg/kg once a day, two weeks 5 mg/kg once a day, and one week 2.5 mg/kg once a day, end of tapering period: T20 wks) and the study ended four weeks after completely stopping treatment (end of study: T24 wks). Plasma exogenous creatinine-iohexol clearance tests (PEC-ICT), kidney biopsies and urine sampling were performed in both groups at baseline, at T16 wks and at T24 wks. Blood and urine samples were additionally collected during treatment after 4 wks (T4 wks) and at T20 wks.

At T16 wks and T24 wks, an ACTH-stimulation test was performed in all dogs by collecting serum for measurement of cortisol before and 60 minutes (min) after intramuscular injection of 0.25 mg of synthethic ACTH (Synacthen, tetracosactide hexa-acetate, Novartis Pharma, Vilvoorde, Belgium). Cortisol concentrations were measured using an immunoassay validated for use in the dog (Architect I 2000 System, Abbott, Waver, Belgium).

### Sampling methods

Morning urine samples were taken by cystocentesis (10 mL, 22 G needle). Urinary dipstick analysis (Combur stick, Roche Diagnostics, Burgess Hill, UK), urine specific gravity (USG), urinary protein-to-creatinine ratio (UPC), sediment analysis and bacterial culture were performed. After centrifugation, the urine supernatant was stored at −80°C until analysis of urinary markers.

### Plasma exogenous creatinine-iohexol clearance test

All dogs were fasted for at least 10 h prior to the test day and fed immediately after ending the sampling period. Water was provided *ad libitum*. Glomerular filtration rate was measured by plasma clearance of exogenous creatinine (Cl_creat_), exo- and endo-iohexol (Cl_exo_ and Cl_endo_), adapted from a protocol previously proposed in cats [31]. Combined IV administration of iohexol and creatinine has already been performed in dogs for renal and plasma clearance procedures [32]. Briefly, a 15 mL baseline blood sample was collected from the jugular vein (22 G needle) for basal creatinine concentration. A 22 G catheter was placed in the cephalic vein, 64.7 mg/kg (0.1 mL/kg) iohexol (Omnipaque 300, GE Healthcare, Amersham Health, Wemmel, Belgium) was injected, followed by 40 mg/kg of a creatinine solution and a timer was started at the end of the injection. Afterwards, dead space in the catheter was rinsed with 3 mL of sodium chloride 0.9%. The catheter was removed and 2.5 mL EDTA blood samples were collected from the jugular vein at 5, 15, 60, 120, 240, 360 and 480 min after injection. Samples were centrifuged within 2 h and stored in aliquots of 300 µL at −20°C until assayed.

Plasma creatinine (Cr) concentrations were analysed using an enzymatic method (Vettest 8008, Idexx, Hoofddorp, The Netherlands). This technique was validated by measuring samples with increasing Cr concentration 4 times per day, on each of 3 consecutive days. The upper limit of quantification was 1202 µmol/L. Within- and between-day coefficients of variation were <3% in the lower, middle, and high concentration range (80, 530 and 1160 µmol/L, respectively), and there was linear correlation between theoretical and measured concentrations within quantification limits. The basal plasma Cr concentration measured on the day of PEC-ICT testing was subtracted from the Cr concentrations measured in the samples from that dog.

Plasma concentrations of iohexol stereo-isomers (exo- and endo-iohexol) were determined by a validated high-performance liquid chromatographic method with ultraviolet detection, as previously described [33]. Percentage of exo- and endo-iohexol measured in the iohexol solution were 83.4% and 16.6%, respectively. This ratio was used to determine the dose of each isomer for clearance calculations. Plasma concentrations below the limit of quantification (LOQ) were not taken into account. The LOQ was 0.43 and 0.06 µg/ml for exo- and endo-iohexol, respectively.

Pharmacokinetic analyses were performed using WinNonlin (WinNonlin version 4.0.1, Pharsight). For clearance calculation, individual plasma data were subjected to noncompartmental analysis, as described by Watson and coworkers [34]. The area under the plasma Cr, exo- and endo-iohexol concentration versus time curve (AUC) was calculated using the trapezoidal rule with extrapolation to infinity. Plasma Cl_creat_, Cl_exo_ and Cl_endo_ were determined by dividing the actually administered dose of exogenous creatinine, exo- and endo-iohexol, respectively, by the corresponding AUC, and indexation to bodyweight (mL/min/kg).

### Serum and urinary renal markers

Serum Cr (sCr) (Vettest 8008, Idexx, Hoofddorp, The Netherlands) and urea (Roche hitachi, Roche diagnostics, Burgess Hill, UK) were measured using an enzymatic method. Urinary protein was determined with a turbidimetric method using benzethonium chloride, and urinary creatinine with a modified Jaffé reaction using picric acid (Roche hitachi, Roche diagnostics, Burgess Hill, UK). As markers of glomerular function, urinary (u) ALB and uIgG concentrations were determined with a commercial canine specific enzyme-linked immunosorbent assay (ELISA) (Immunology Consultants Laboratory, Newberg, OR, USA). As an indication of tubular function, uRBP was determined with a human ELISA (Immunology Consultants Laboratory, Newberg, OR, USA) and uNAG activity using a colorimetric assay (β-N-Acetylglucosaminidase Assay kit, Sigma-Aldrich, St Louis, MO, USA). All assays had been previously described and validated in our laboratory [22], [35]. Within- and between-day coefficients of variation were 5.2 and 12.0% for the uALB ELISA, 6.1 and 7.7% for the uIgG ELISA, 4.3 and 5.5% for the uRBP ELISA, and 4.9 and 8.1% for the uNAG colorimetric assay. Because spot urine samples were used, urinary concentration of each marker was indexed to urinary creatinine (c) and expressed as a ratio: uALB/c, uIgG/c, uRBP/c and uNAG/c [36], [37].

### Kidney biopsies

Coagulation was checked in all dogs by measurement of buccal mucosal bleeding time, and activated partial thromboplastin and prothrombin time (CA 7000, Siemens, Brussels, Belgium). Ultrasound guided percutaneous kidney biopsies were taken under general anesthesia at T0, T16 wks and T24 wks. Dogs were premedicated intravenously (IV) with a combination of acepromazine (0.01 mg/kg) (Placivet, Codiphar, Wommelgem, Belgium) and butorphanol (0.01 mg/kg) (Dolorex, Intervet NV, Mechelen, Belgium). Afterwards, dogs received IV diazepam (0.2 mg/kg) (Valium, Rosch, Brussels, Belgium) immediately followed by propofol (Propovet, Abbott Animal Health, Queenborough, UK) to effect until endotracheal intubation could be performed. Anesthesia was maintained with isoflurane (2% vaporized in 100% oxygen) (Isoflo, Abbott Animal Health, Queenborough, UK).

Ultrasound guided kidney biopsies were taken by an experienced radiologist, according to a previously described technique [38]. Large-bore 14-gauge needles with 9 cm length and 20 mm specimen notch (Vet-CoreTM, Surgivet, USA) were used to take two consecutive biopsies >10 mm long (or three biopsies <10 mm long) from the left kidney. Biopsy cores were collected and transported according to a standardized protocol adapted from Lees and co-workers [39]. Specimens were transversely divided in three parts and transferred into a standard container with 3% glutaraldehyde for EM within 5 min after collection, in 10% buffered formalin for LM and in Michel's transport medium for IF. Biopsies were cooled and transported to a nephropathology service within 24 h.

Three µm sections from each biopsy sample were stained with hematoxylin and eosin (HE), periodic acid-Schiff (PAS), Masson's trichrome and periodic acid methenamine silver. All biochemical stains were performed according to standard procedures [39]. Slides were evaluated using light microscopy for presence of glomerular, tubular, interstitial and vascular lesions, using a tie-grade system from 1 to 4 (1 = rare lesion, 2 = mild, 3 = moderate, 4 = severe).

For direct immunofluorescence (IF) examination, fresh unfixed renal specimens were embedded in OCT compound, snap-frozen in liquid nitrogen and stored at −80°C. Subsequently, 5 µm thick sections were fixed with acetone for 15 min. After washing with PBS (two passages), slides were incubated with FITC-labelled sheep anti-dog IgG, goat anti-dog IgM, goat anti-dog IgA, goat anti-dog C3 (all 4 from Bethyl Laboratories Inc., Montgomery, TX, USA), rabbit anti-human κ light chain, rabbit anti-human λ light chain, and rabbit anti-human complement factor C1Q (all 3 from Dako, Glostrup, Denmark). Primary antibodies were omitted as negative controls and substituted with PBS [40]. Fluorescence findings were classified according to the pattern seen on immunofluorescent examination (granular or linear), which included the localization of the deposit (mesangium, GBM, tubules and vessels), the distribution (focal, diffuse, segmental and global) and the intensity.

For transmission electron microscopy (EM), the tissue was post-fixed in 2% osmium (in distilled water) for 1.5 h, dehydrated in graded acetone and embedded in epon. Semi-thin sections were stained with azure methylene blue. Ultrathin sections were counterstained with uranyl acetate and lead citrate. For ultrastructural studies, a Philips EM 420 was used. All histological sections were examined in a blinded fashion by three pathologists.

### Statistical analysis

Analyses were performed with a commercial software program (Systat, version 12.00.08). Level of significance was set at 5%. A general linear model was used to test the effect of HC treatment, time point and their interaction between the control and the HC group. When a statistically significant interaction between treatment and time point was detected, a post hoc hypothesis test was performed to compare the two groups at each period.

## Results

### Induction of and recovery from hypercortisolism

All dogs assigned to the HC group developed clinical signs of hypercortisolism, such as polyuria, polydipsia and skin changes (atrophy of abdominal skin with phlebectasia, no regrowth of clipped haircoat, comedones, ecchymose) and complementary laboratory changes (increased alkaline phosphatase and alanine aminotransferase, ALT). Additionally, some dogs also developed muscle atrophy and abdominal distention, but no significant changes in bodyweight were detected throughout the study. At T16 wks, 36 h after HC withdrawal, all dogs in the HC group failed to respond adequately to ACTH-stimulation; median (range) serum cortisol concentrations before and after ACTH-injection were 18 nmol/L (6–30 nmol/L) and 25 nmol/L (17–86 nmol/L). At T16 wks serum ALT was significantly increased in the HC group compared to the control group (*p*<0.001).

At T24 wks, 4 weeks after complete HC withdrawal, clinical signs were resolved, except for skin changes in some dogs, and laboratory changes returned to baseline (alkaline phosphatase and ALT within reference interval). Response to an ACTH-stimulation test was within normal range in all dogs; median (range) serum cortisol concentrations before and after ACTH-injection were 32 nmol/L (25–60 nmol/L) and 330 nmol/L (246–516 nmol/L).

### Plasma exogenous creatinine-iohexol clearance test

**Figure 1:**
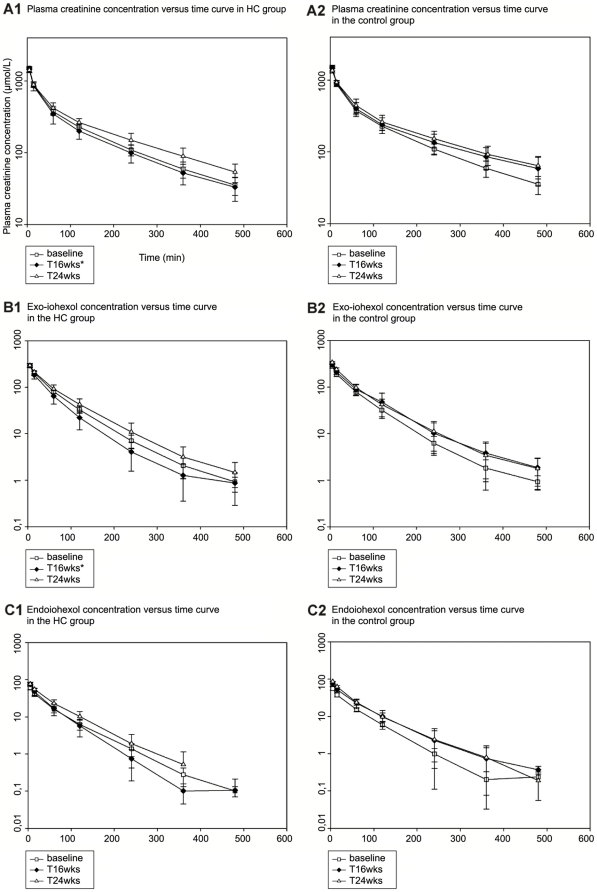
Plasma concentration versus time curves. Plasma creatinine (A1, A2), exo-iohexol (B1, B2) and endo-iohexol concentration (C1, C2) versus time curves in the hydrocortisone (HC) group (6 dogs) and the control group (5 dogs) at T0, T16 wks and T24 wks. Mean ± standard deviation is displayed. The asterisk indicates a statistically significant difference (*p*<0.001) in plasma clearance between groups at T16 wks, for creatinine and exo-iohexol clearance.

As presented in Table 1, plasma Cl_creat_ and Cl_exo_ were significantly higher in the HC group than in the control group at T16 wks (*p*<0.001). There was no significant difference between groups either at baseline or at T24 wks. Plasma Cl_endo_ was not significantly different between groups at any time point (*p* = 0.21). Figure 1 shows the plasma creatinine (A), exo-iohexol (B) and endo-iohexol concentration (C) versus time curves in the control and HC groups at baseline, T16 wks and T24 wks.

**Table 1:** Glomerular filtration rate and routine renal markers in the control and hydrocortisone group.

Renal Marker	T0		T16 wks		T24 wks	
	Controls	HC	Controls	HC	Controls	HC
Cl_creat_ (mL/min/kg)	3.3 (2.8–3.7)	3.3 (2.6–3.7)	3.0 (2.1–3.4)	3.7 (2.5–4.2)*	2.8 (2.0–3.0)	2.7 (2.3–3.3)
Cl_exo_ (mL/min/kg)	2.9 (2.7–3.6)	3.2 (2.5–3.7)	2.9 (2.1–3.4)	4.0 (2.5–4.5)*	2.9 (2.1–3.1)	2.8 (2.0–3.3)
Cl_endo_ (mL/min/kg)	3.1 (2.8–3.9)	2.9 (2.3–4.5)	2.5 (1.6–2.9)	3.2 (1.8–3.9)	2.3 (1.8–2.4)	2.3 (1.6–2.7)
sCr (µmol/L)	72 (58–92)	80 (75–84)	86 (75–113)	88 (68–111)	90 (76–119)	90 (68–107)
urea (mmol/L)	13.1 (7.3–17.2)	11.0 (9.9–14.6)	10.3 (8.1–25.9)	9.2 (6.0–20.4)	11.8 (7.7–27.4)	10.8 (8.1–16.3)
USG	1.018	1.024	1.018	1,003	1,012	1.019
	(1.008–1.035)	(1.012–1.050)	(1.011–1.020)	(1.000–1.013)	(1.006–1.050)	(1.007–1.029)
UPC	1.77 (0.12–10)*	0.18 (0.13–1.56)	1.93 (0.11–6.55)	3.04 (0.28–6.19)	1.95 (0.15–6.67)*	0.16 (0.02–0.33)

T0, baseline; T16 wks, end of the 16 weeks hydrocortisone treatment period; T24 wks, 4 weeks after complete cessation of hydrocortisone treatment; HC, hydrocortisone group; controls, control group; Cl_creat_, plasma clearance of creatinine; Cl_exo_, plasma clearance of exo-iohexol, Cl_endo_, plasma clearance of endoiohexol; sCr, serum creatinine; USG, urinary specific gravity; UPC, urinary protein-to-creatinine ratio. The asterisk indicates in which group results are significantly increased (*p*<0.05) compared to the other group at that timepoint.

### Serum and urinary renal markers

**Figure 2:**
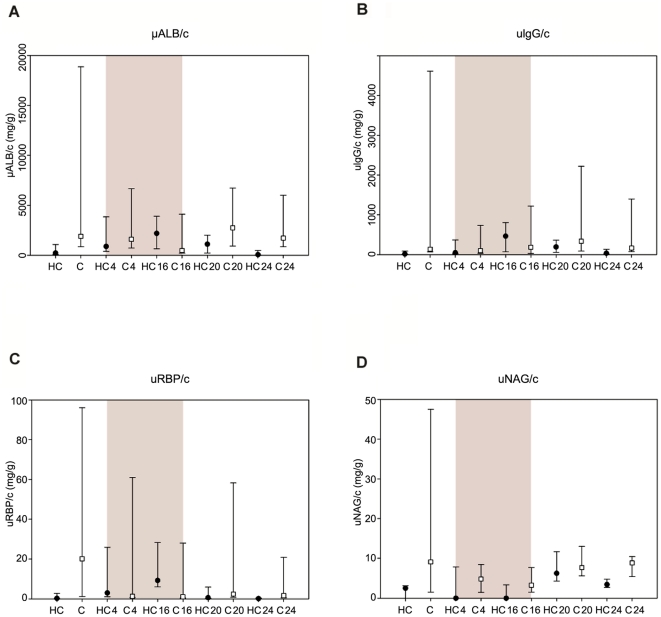
Urinary markers. Median and 75-25^th^ percentiles of urinary albumin-to-creatinine ratio (uALBc) (A), immunoglobulin G-to-creatinine ratio (uIgG/c) (B), retinol-binding protein-to-creatinine ratio (uRBP/c) (C) and N-acetyl-β-D-glucosaminidase ratio (uNAG/c) (D) at T0, T4 wks, T16 wks, T20 wks and T24 wks in the hydrocortisone (HC) group (black circles) and in the control (C) group (white squares). The grey area indicates the hydrocortisone administration period.

As shown in Table 1, no significant differences were detected between groups for sCr (*p* = 0.74), urea concentration (*p* = 0.32) and USG (*p* = 0.30) throughout the study, despite a decrease of USG during treatment in the HC group. Urinary protein-to-creatinine ratio was significantly higher in the control group than in the HC group (*p* = 0.003) at baseline, T4 wks, T20 wks and T24 wks, but not at T16 wks. Figure 2 presents the median and 75-25^th^ percentiles of UPC, uALB/c, uIgG/c, uRBP/c and uNAG/c in the HC and control groups before, during and after HC administration, with the grey area representing the treatment period. Differences between HC and control groups in glomerular markers uALB/c (*p* = 0.05) and uIgG/c (*p* = 0.26) did not reach statistical significance, although they progressively increased within the HC group during treatment and decreased afterwards. Also for tubular markers uRBP/c (*p* = 0.07) and uNAG/c (*p* = 0.20), there was no significant difference between the HC and control groups at any time point, despite the fact that uRBP/c seemed to increase within the HC group, with highest concentrations at T16 wks, and declined again after discontinuation of HC.

### Kidney histopathology

Most important light microscopic findings in HC and control dogs are presented in Table 2 and 3. Affected dogs in both groups and the score ranges of severity at different time points are mentioned.

**Table 2:** Glomerular lesions in the hydrocortisone (n = 6 dogs) and control group (n = 5 dogs).

Dog number	Global endocap. hypercellularity			Prim. mesang. hypercellularity			Diffuse glomerulosclerosis			Global glomerulosclerosis			Number of obsolescent glomeruli		
	T0	T16 wks	T24 wks	T0	T1 wks	T2 wks	T0	T1 wks	T24 wks	T0	T1 wks	T24 wks	T0	T16 wks	T24 wks
**HC group**															
Dog 1	0	0	2	0	0	2	2	2	4	2	3	2	0	5/94	5/39
Dog 2	0	0	0	0	0	2	0	0	4	0	0	2	0	0	4/63
Dog 3	2	2	2	2	3	2	0	2	4	0	2	3	0	3/55	9/90
Dog 4	0	0	0	0	0	0	1	1	3	1	1	2	0	3/52	2/26
Dog 5	0	0	0	0	2	1	2	2	3	2	2	2	0	4/56	4/35
Dog 6	0	0	2	0	0	2	2	2	3	2	0	2	0	0	4/37
**Control group**															
Dog 7	2	2	2	3	3	2	3	3	4	3	3	2	0	6/44	6/83
Dog 8	0	2	2	0	2	2	2	4	3	2	4	2	5/12	18/79	4/19
Dog 9	0	0	0	0	0	0	0	0	2	0	0	2	1/22	3/47	5/42
Dog 10	3	2	2	2	3	3	3	2	2	3	2	2	6/39	8/57	4/46
Dog 11	0	0	0	0	0	0	0	2	2	0	1	1	0	2/69	1/45

Endocap., endocapillary; Prim.mesang., primary mesangial; T0, baseline; T16 wks, end of the 16 weeks hydrocortisone treatment period; T24 wks, 4 weeks after complete cessation of hydrocortisone treatment; HC, hydrocortisone. Score: 1 = rare lesion, 2 = mild, 3 = moderate, 4 = severe.

**Table 3:** Tubular and interstitial lesions in the hydrocortisone (n = 6 dogs) and control group (n = 5 dogs).

Dog number	Tubular atrophy			Tubular cell pigment			Interstitial fibrosis			Interstitial inflammation		
	T0	T16 wks	T24 wks	T0	T16 wks	T24 wks	T0	T16 wks	T24 wks	T0	T16 wks	T24 wks
**HC group**												
Dog 1	0	3	2	2	2	2	0	2	2	0	2	2
Dog 2	0	0	0	1	1	1	0	0	2	1	0	2
Dog 3	0	2	4	1	1	1	0	1	4	1	2	3
Dog 4	0	3	3	2	2	2	0	0	2	0	2	2
Dog 5	0	0	0	2	2	2	0	0	0	0	1	0
Dog 6	0	0	0	1	0	2	0	0	0	0	0	1
**Control group**												
Dog 7	1	2	0	0	2	1	0	2	2	1	3	2
Dog 8	3	3	2	2	2	2	2	2	2	2	3	2
Dog 9	0	2	2	0	0	2	0	2	2	1	2	2
Dog 10	2	2	2	2	2	2	2	0	2	2	2	2
Dog 11	0	2	0	2	2	2	0	2	0	1	2	2

T0, baseline; T16 wks, end of the 16 weeks hydrocortisone treatment period; T24 wks, 4 weeks after complete cessation of hydrocortisone treatment; HC, hydrocortisone. Score:

1 = rare lesion, 2 = mild, 3 = moderate, 4 = severe.

**Figure 3:**
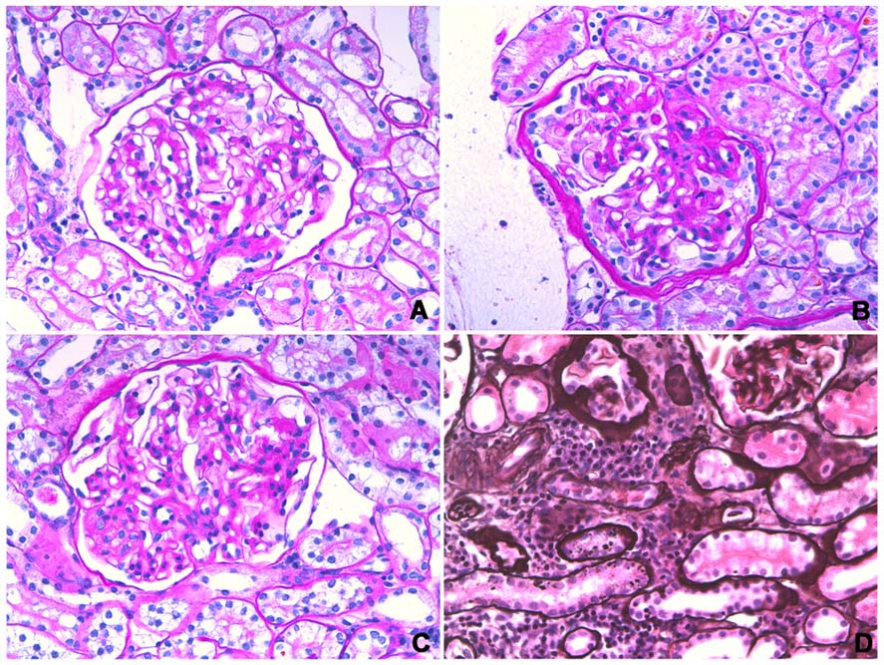
Light microscopic lesions. (A) Glomerulus, Dog 11 at T0. Early glomerular lesions including minimal increase of mesangial matrix. Periodic acid-Schiff staining (×400); (B) Glomerulus, Dog 11 at T16 weeks. Moderate hypercellularity of mesangial and endothelial cells and one synechia. There is segmental to global increase of mesangial matrix and Bowman's capsule is symmetrically thickened with proliferation of parietal epithelial cells. Periodic acid-Schiff staining (×400); (C) Glomerulus, Dog 11 at T24 weeks. Moderate hypercellularity of mesangial and endothelial cells associated with global increase of mesangial matrix. Visceral epithelial cells are hypertrophic. Periodic acid-Schiff staining (×400); (D) Tubulo-interstitium, Dog 11 at T24 weeks. Moderate inflammation widely separating tubules and atrophic tubules. Periodic acid-methenamine silver staining (×400).

**Figure 4:**
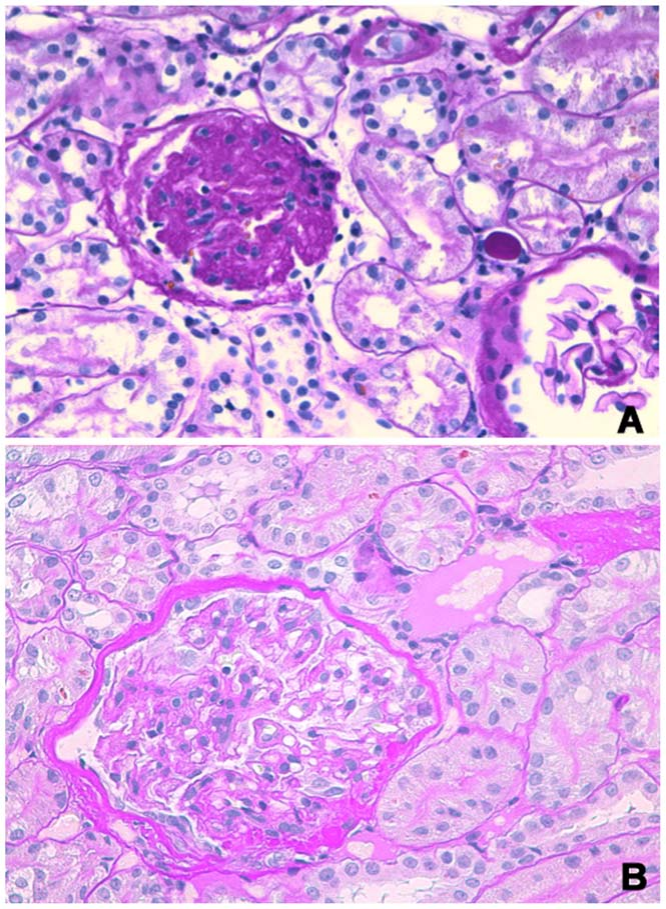
Glomerular lesions. (A) Glomerulus, Dog 8 at T16 weeks. Obsolescent glomerulus. Periodic acid-Schiff staining (×400). (B) Glomerulus, Dog 5 at T24 weeks. Accentuated lobulation, increase of mesangial and endothelial cellularity of the tuft, and synechiae. Splitting of Bowman's capsule is visible. Periodic acid-Schiff staining (×400).

Commonly observed glomerular lesions were global endocapillary and mesangial hypercellularity, with increase of mesangial matrix. Increase of mesangial matrix progressed in both the HC and control group at T16 wks and T24 wks, with an increasing number of affected dogs and highest scores at T24 wks (Figure 3a, 3b and 3c). Obsolescent glomeruli were detected in one control dog at T0, in 4 HC dogs and all control dogs at T16 wks, and in all dogs at T24 wks (Figure 4a). Asymmetrical thickening and splitting of Bowman's capsule was most pronounced in two control dogs especially at T24 wks, whereas only rare to mild changes were seen in the HC group, mainly at T16 wks (Figure 4b). Tubular lesions included tubular atrophy, tubular macro- and micro-vesiculation and cellular pigment. In the HC group there was no tubular atrophy at T0, whereas mild to severe atrophy was detected in three dogs at T16 wks and T24 wks. Rare to moderate tubular atrophy was present throughout the study in three to five control dogs. At every time point, up to five HC dogs and all control dogs had rare to moderate interstitial inflammation (Figure 3d). Rare to mild interstitial fibrosis was found in up to three HC dogs at T16 wks and T24 wks, and severe fibrosis in one HC dog at T24 wks. Up to four control dogs had rare to mild fibrosis at all time points.

**Figure 5:**
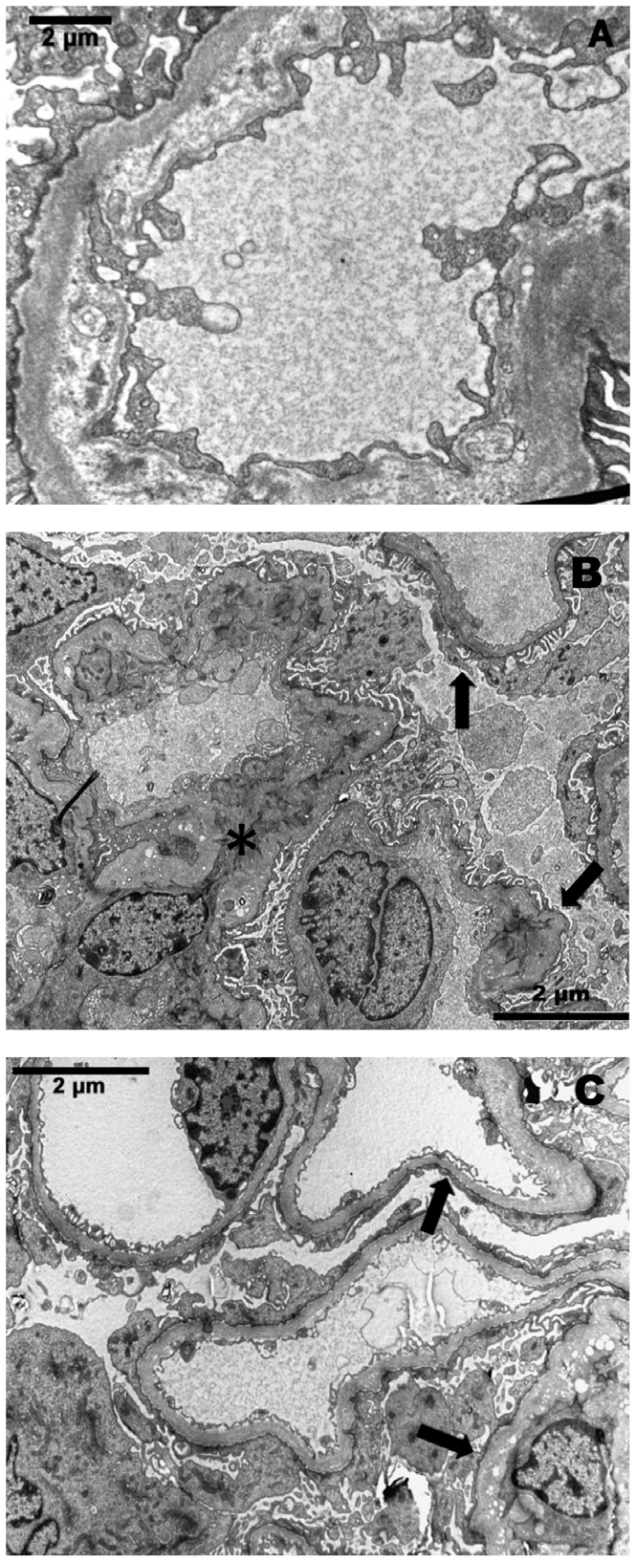
Electron microscopic glomerular lesions. (A) Glomerulus, Dog 10 at T16 weeks. Sub-endothelial widening and rarefaction of the glomerular basement membrane associated with foot process effacement. EM ×5000; (B) Glomerulus, Dog 9 at T24 weeks. Multifocal foot process effacement (arrow) and endothelial cell swelling with reduction of the capillary lumens and mesangial interposition (asterisk). EM ×4000; (C) Glomerulus, Dog 11 at T24 weeks. Diffuse and severe foot process effacement (arrows). EM ×4000.

Ultrastructural lesions included changes of the glomerular basement membrane (GBM) such as thickening, rarefaction, wrinkling and irregular jagged profile (Figure 5a). At T0, EM detected GBM changes in only 2/6 HC dogs, but they were prominent in the entire HC group at T16 wks and T24 wks. In the control group mild to moderate GBM lesions were found at all time points. Multifocal to diffuse podocyte foot process fusion was found in 5/6 HC dogs and all five control dogs at every time point (Figure 5b and 5c). Degenerative tubular cells and/or degenerative changes in podocytes, endothelial and mesangial cells were detected in dogs from both groups and were most pronounced at T24 wks.

Due to a technical error concerning the Michel's transport medium, biopsies could not be evaluated with IF at T0. Immunofluoresence staining at T16 wks and T24 wks was unremarkable with coarse, segmental deposits of Ig G (1+ to 2+) in the capillary wall in 1/6 HC dogs and 2/5 control dogs at T16 wks. Mesangial Ig M deposits (1+ to 2+) were detected in 4/6 HC dogs and 3/5 control dogs at T16 wks, and in one control dog at T24 wks. Coarse λ light chain deposits (1+ to 2+) were seen in the capillary walls and mesangium of one dog from each group at T16 wks, and in one control dog at T24 wks. One dog from the HC group had 1+ coarse, diffuse, segmental deposits of C3 at T24 wks.

In summary, glomerular, tubular and interstitial lesions presented in Table 2 and 3 progressed over time in both groups, with most severe lesions detected at T24 wks. At T16 wks, a marked increase was seen in the number of HC dogs with obsolescent glomeruli and tubular atrophy compared to baseline. No significant vascular changes (hypertrophic and hyaline arteriosclerosis, arteritis, fibrinoid necrosis, thrombosis) were present at baseline or at any of the other time points.

## Discussion

This study demonstrates that HC administration reversibly increases GFR in aged Beagle dogs. Plasma Cl_creat_ and Cl_exo_ were both significantly increased in the HC group at T16 wks, but no longer differed between groups eight weeks after tapering and cessation of treatment. This is in agreement with previous reports showing that synthetic GC augment GFR in dogs, with an increase in renal plasma flow (RPF), renal vasodilation and a decrease in filtration fraction (FF) [5], [41]. The mechanism for this increase is most likely at least partly species-specific, since GC also increase GFR in humans, but with little effect on RPF and an increase instead of a decrease in FF [42]. Suggested mechanisms include hemodynamic factors, interaction of GC with vaso-active hormones and catabolic effects leading to an increase in plasma amino acids, which in turn augments RPF and GFR [43], [44]. Glucocorticoids can also mediate GFR changes through an increased blood pressure, leading to a higher intraglomerular pressure, although they do not always cause hypertension in dogs [10]. However, blood pressure measurements were not available in the current study.

Although all dogs included in the study were clinically healthy with no abnormalities on hematology, biochemistry profile and abdominal ultrasound, two dogs from the HC group and four dogs from the control group had an increased UPC at baseline. Proteinuria, which did not progress over a two year follow-up period, has been previously described in otherwise healthy Beagles by Stuart and coworkers [45]. In the latter study, LM and EM changes were similar to our results and degree of proteinuria correlated with the severity of glomerular lesions [45]. In the present study, dogs with most severe glomerular changes also had the highest UPC (n° 3: 1.56, n° 7: 1.77, n° 8: 10 and n° 10: 6.4; reference range <0.5). The dog with the highest UPC had severe glomerulosclerosis and had the highest percentage of obsolescent glomeruli throughout the study. Pre-existing renal lesions and proteinuria, independent of GC administration, in these healthy aged Beagle dogs raise questions for interpretation of similar findings in dogs with glomerular diseases. Moreover, this important observation has to be kept in mind when using Beagle dogs as a model for human renal disease or toxicological studies [46].

Proteinuria was significantly higher in the control group than in the HC group at all time points, except for T16 wks. This could be caused by an increase in UPC in the HC group at the end of the treatment period. Table 1 and Figure 2 indicate that UPC was increased in the HC group at T16 wks and progressively decreased again at T20 wks and T24 wks. Figure 2 also suggests an increase followed by a decline within the HC group for uALB/c, uIgG/c and uRBP/c, but not for uNAG/c. Nevertheless, dogs with the highest UPC also had the highest uNAG/c, which could be an indication of increased tubular lysosomal protein degradation. Interestingly, changes in the lysosomal degradation pattern with altered distribution of lysosomes from the perinuclear to the apical tubular cell region were also detected in proteinuric X-linked Alport syndrome affected dogs [24].

Possible mechanisms causing glomerular proteinuria include glomerular hypertension and structural alterations of the glomerular barrier [47]. A GC-induced increase in GFR together with hemodynamic alterations could augment glomerular pressure and contribute to proteinuria. This hypothesis is supported by the parallel increase of both GFR and proteinuria at the end of the HC treatment period and their simultaneous decline after cessation of treatment in the HC group. Structural alterations that may contribute to proteinuria were glomerulosclerosis, podocyte foot process effacement, and GBM changes such as wrinkling, thickening, delamination and rarefaction. Whereas proteinuria and hyperfiltration were rapidly reversible after cessation of treatment in the HC group, the structural changes seemed to persist at T24 wks. Possible explanations for this are three-fold: 1) the lesions are not related to GC administration, but are age-dependent and continue their physiological progression over time, 2) the lesions are GC-induced and there is a longer lag-time than eight weeks between tapering and cessation of HC administration and normalization of structural changes, and 3) the lesions are irreversible. Because it was not possible to repeat kidney biopsies at a later time in the current Beagle colony, the present study cannot confirm or dispute any of these hypotheses. It has been previously demonstrated that serial cortical biopsies can be obtained with minimal risk of inducing changes that might be confused with those of a progressive renal disease [48]. Moreover, ultrasound examination revealed healing of the needle tract lesions within 2 to 3 weeks post-biopsy in both the HC and control group, as also reported in literature [49]. Therefore, it is highly unlikely that the renal lesions observed at T24 wks are a consequence of repeated biopsy procedures.

At baseline, many dogs had mild to moderate pre-existing renal lesions. Glomerulosclerosis has been described to be age-related in dogs and in people [46], [50]. Tubular atrophy, and interstitial inflammation and fibrosis have also been shown to increase with aging in both species [46], [51], [52]. The brownish cellular pigment detected throughout the study in many dogs is most likely lipofuscin accumulation, which again may be an age-related phenomenon as previously suggested in rats and dogs [51], [53]. Interestingly, no vascular morphological changes were found throughout the current study period in any of the groups, whereas in people vascular changes are part of age-related renal lesions [54], [55].

At the end of the HC administration period, most striking LM findings in the HC group, not detected at baseline, were obsolescent glomeruli and tubular atrophy. However, there was a higher prevalence of renal lesions at baseline than expected and glomerular, tubular and interstitial changes also progressed in the control group. Therefore, it is not possible to separate HC effects from progression of pre-existing renal lesions. It is possible that HC administration accelerated age-related glomerulosclerotic and tubular changes in the HC group, but definitive conclusions cannot be drawn from these data. Another limitation of the study is the lack of IF examination at baseline. However, IF findings at T16 wks and T24 wks were either mild or non-specific. For example, the mesangial IgM deposits detected in some HC and control dogs at T16 wks and in one control dog at T24 wks were probably due to entrapment of IgM in sclerotic glomeruli.

In conclusion, this study shows that HC administration reversibly increases GFR, with a similar trend for proteinuria. Results indicate that clinically healthy aged Beagle dogs have renal lesions and proteinuria, which could have implications for experimental or toxicological studies. Although further research is needed to elucidate whether morphological renal changes are GC-induced, hyperfiltration and proteinuria are, and warrant attention to kidney function in human and canine patients with Cushing's syndrome or receiving exogenous GC.

## References

[1] Goy-Thollot I, Pechereau D, Keroack S, Dezempte JC, Bonnet JM (2002). Investigation of the role of aldosterone in hypertension associated with spontaneous pituitary-dependent hyperadrenocorticism in dogs.. J Small Anim Pract.

[2] Hurley KJ, Vaden SL (1998). Evaluation of urine protein content in dogs with pituitary-dependent hyperadrenocorticism.. J AmVet Med Assoc.

[3] Koh JM, Kim JY, Chung YE, Park JY, Shong YK (2000). Increased urinary albumin excretion in Cushing's syndrome: remission after correction of hypercortisolaemia.. Clin Endocrinol.

[4] Haentjens P, De Meirleir L, Abs R, Verhelst J, Poppe K (2005). Glomerular filtration rate in patients with Cushing's disease: a matched case-control study.. Eur J Endocrinol.

[5] Hall JE, Morse CL, Smith MJ, Young DB, Guyton AC (1980). Control of arterial pressure and renal function during glucocorticoid excess in dogs.. Hypertension.

[6] van Acker BA, Prummel MF, Weber JA, Wiersinga WM, Arisz L (1993). Effect of prednisone on renal function in man.. Nephron.

[7] de Bruin C, Meij BP, Kooistra HS, Hanson JM, Lamberts SWJ (2009). Cushing's Disease in Dogs and Humans.. Horm Res.

[8] Orth DN (1995). Cushing's syndrome.. N Engl J Med.

[9] Rijnberk A, der Kinderen PJ, Thijssen JH (1968). Spontaneous hyperadrenocorticism in the dog.. J Endocrinol.

[10] Schellenberg S, Mettler M, Gentilini F, Portmann R, Glaus TM (2008). The effects of hydrocortisone on systemic arterial blood pressure and urinary protein excretion in dogs.. J Vet Intern Med.

[11] Waters CB, Adams LG, ScottMoncrieff JC, DeNicola DB, Snyder PW (1997). Effects of glucocorticoid therapy on urine protein-to-creatinine ratios and renal morphology in dogs.. J Vet Intern Med.

[12] Finco DR, Brown SA, Crowell WA, Brown CA, Barsanti JA (1994). Effects of aging and dietary protein intake on uninephrectomized geriatric dogs.. Am J Vet Res.

[13] Nishida E, Yamanouchi J, Ogata S, Itagaki S, Doi K (1996). Age-related histochemical and ultrastructural changes in renal glomerular mesangium of APA hamsters.. Exp Anim.

[14] Pugliese A, Gruppillo A, Di Pietro S (2005). Clinical nutrition in gerontology: chronic renal disorders of the dog and cat.. Vet Res Commun.

[15] Silva FG (2005). The aging kidney: a review – part I.. Int Urol Nephrol.

[16] Silva FG (2005). The aging kidney: a review–part II.. Int Urol Nephrol.

[17] Esposito C, Plati A, Mazzullo T, Fasoli G, De Mauri A (2007). Renal function and functional reserve in healthy elderly individuals.. J Nephrol.

[18] Fuiano G, Sund S, Mazza G, Rosa M, Caglioti A (2001). Renal hemodynamic response to maximal vasodilating stimulus in healthy older subjects.. Kidney Int.

[19] Raila J, Forterre S, Kohn B, Brunnberg L, Schweigert FJ (2003). Effects of chronic renal disease on the transport of vitamin A in plasma and urine of dogs.. Am J Vet Res.

[20] Yalcin A, Cetin M (2004). Electrophoretic separation of urine proteins of healthy dogs and dogs with nephropathy and detection of some urine proteins of dogs using immunoblotting.. Rev Med Vet.

[21] Zini E, Bonfanti U, Zatelli A (2004). Diagnostic relevance of qualitative proteinuria evaluated by use of sodium dodecyl sulfate-agarose gel electrophoresis and comparison with renal histologic findings in dogs.. Am J Vet Res.

[22] Smets PMY, Meyer E, Maddens BEJ, Duchateau L, Daminet S (2010). Urinary markers in healthy young and aged dogs and dogs with chronic kidney disease.. J Vet Intern Med.

[23] Zaragoza C, Barrera R, Centeno F, Tapia JA, Mane MC (2003). Characterization of renal damage in canine leptospirosis by sodium dodecyl sulphate-polyacrylamide gel electrophoresis (SDS-PAGE) and Western blotting of the urinary proteins.. J Comp Pathol.

[24] Vinge L, Lees GE, Nielsen R, Kashtan CE, Bahr A (2010). The effect of progressive glomerular disease on megalin-mediated endocytosis in the kidney.. Nephrol Dial Transplant.

[25] Raila J, Brunnberg L, Schweigert FJ, Kohn B (2010). Influence of kidney function on urinary excretion of albumin and retinol-binding protein in dogs with naturally occurring renal disease.. Am J Vet Res.

[26] Maddens B, Daminet S, Smets P, Meyer E (2010). Escherichia coli pyometra induces transient glomerular and tubular dysfunction in dogs.. J Vet Intern Med.

[27] Castagnaro M (1987). Histopathological and ultrastructural studies of kidney lesions in a case of Cushing's syndrome in a dog.. Schweiz Arch Tierheilkd.

[28] Hsieh CK, Hsieh YP, Wen YK, Chen ML (2007). Focal segmental glomerulosclerosis in association with Cushing's disease.. Clin Nephrol.

[29] Stuart S, Chandran PKG (2001). Oncogenic Cushing's syndrome and nephrotic syndrome in the same patient.. Am J Kidney Dis.

[30] Tatsumi T, Morishima T, Watarai T, Kubota M, Kodama M (1995). Recurrent Cushing's disease associated with nephrotic syndrome.. Intern Med.

[31] Le Garreres A, Laroute V, De La Farge F, Boudet KG, Lefebvre HP (2007). Disposition of plasma creatinine in non-azotaemic and moderately azotaemic cats.. J Feline Med Surg.

[32] Mathieu M, Motte S, Ray L, Pensis A, Jespers P (2006). Effects of ramipril on renal function during progressive overpacing-induced heart failure in dogs.. Am J Vet Res.

[33] van Hoek I, Vandermeulen E, Duchateau L, Lefebvre HP, Croubels S (2007). Comparison and reproducibility of plasma clearance of exogenous creatinine, exo-iohexol, endo-iohexol, and Cr-51-EDTA in young adult and aged healthy cats.. J Vet Intern Med.

[34] Watson AD, Lefebvre HP, Concordet D, Laroute V, Ferre JP (2002). Plasma exogenous creatinine clearance test in dogs: comparison with other methods and proposed limited sampling strategy.. J Vet Intern Med.

[35] Maddens BE, Daminet S, Demeyere K, Demon D, Smets P (2010). Validation of immunoassays for the candidate renal markers C-reactive protein, immunoglobulin G, thromboxane B(2) and retinol binding protein in canine urine.. Vet Immunol Immunopathol.

[36] Grauer GF, Thomas CB, Eicker SW (1985). Estimation of quantitative proteinuria in the dog, using the urine protein-to-creatinine ratio from a random, voided sample.. Am J Vet Res.

[37] Torng S, Rigatto C, Rush DN, Nickerson P, Jeffery JR (2001). The urine protein to creatinine ratio (P/C) as a predictor of 24-hour urine protein excretion in renal transplant patients.. Transplantation.

[38] Vaden SL (2005). Renal biopsy of dogs and cats.. Clin Tech Small Anim Pract.

[39] Lees GE, Cianciolo RE, Clubb FJ (2011). Renal biopsy and pathologic evaluation of glomerular disease.. Top Companion Anim Med.

[40] Aresu L, Pregel P, Bollo E, Palmerini D, Sereno A (2008). Immunofluorescence staining for the detection of immunoglobulins and complement (C3) in dogs with renal disease.. Vet Rec.

[41] Kubota E, Hayashi K, Matsuda H, Honda M, Tokuyama H (2001). Role of intrarenal angiotensin II in glucocorticoid-induced renal vasodilation.. Clin Exp Nephrol.

[42] Connell JMC, Whitworth JA, Davies DL, Lever AF, Richards AM (1987). Effects of ACTH and cortisol administration on blood-pressure, electrolyte metabolism, atrial-natriuretic-peptide and renal-function in normal man.. J Hypertens.

[43] Baylis C, Handa RK, Sorkin M (1990). Glucocorticoids and control of glomerular filtration rate.. Semin Nephrol.

[44] Manning RD (1987). Renal hemodynamic, fluid volume, and arterial pressure changes during hyperproteinemia.. Am J Physiol.

[45] Stuart BP, Phemister RD, Thomassen RW (1975). Glomerular lesions associated with proteinuria in clinically healthy dogs.. Vet Pathol.

[46] Pomeroy MJ, Robertson JL (2004). The relationship of age, sex, and glomerular location to the development of spontaneous lesions in the canine kidney: analysis of a life-span study.. Toxicol Pathol.

[47] Lees GE, Brown SA, Elliott J, Grauer GE, Vaden SL (2005). Assessment and management of proteinuria in dogs and cats: 2004 ACVIM Forum Consensus Statement (small animal).. J Vet Intern Med.

[48] Groman RP, Bahr A, Berridge BR, Lees GE (2004). Effects of serial ultrasound-guided renal biopsies on kidneys of healthy adolescent dogs.. Vet Radiol Ultrasound.

[49] Haers Haers H, Smets P, Pey P, Piron K, Daminet S (2011). Contrast harmonic ultrasound appearance of consecutive percutaneous renal biopsies in dogs.. Vet Radiol Ultrasound.

[50] Kaplan C, Pasternack B, Shah H, Gallo G (1975). Age-related incidence of sclerotic glomeruli in human kidneys.. Am J Pathol.

[51] Heiene R, Kristiansen V, Teige J, Jansen JH (2007). Renal histomorphology in dogs with pyometra and control dogs, and long term clinical outcome with respect to signs of kidney disease.. Acta Vet Scand.

[52] Lindeman RD (1986). The aging kidney.. Compr Ther.

[53] Ikeda H, Tauchi H, Sato T (1985). Fine structural analysis of lipofuscin in various tissues of rats of different ages.. Mech Ageing Dev.

[54] Martin JE, Sheaff MT (2007). Renal ageing.. J Pathol.

[55] McLachlan MS, Guthrie JC, Anderson CK, Fulker MJ (1977). Vascular and glomerular changes in the ageing kidney.. J Pathol.

